# An Account of an Osseous Deposit in the Dura Mater

**Published:** 1843-08

**Authors:** J. W. Stout

**Affiliations:** Nashville, Tennessee


Art. IV.—*An Account of an Osseous Deposit in the Dura Mater.*—Read before the Medical Society of Tennessee. By J. W. Stout, M.D., of Nashville, Tennessee.

The deposit occurred in a negro man named Jacob, who was executed for the murder of his master. He was aged about twenty-seven years, of very large muscular development, and excellent health; was never known to have any affection of his head either mental or physical; on the contrary he was considered more intelligent than country negroes are. His habits were of a temperate order; he is said to have been very irritable and remarkably free from fear when ex cited; indeed he met his fate with almost Socratic fortitude. From a small boy he was remarkably cruel, fond of inflicting pain upon animals, and habitually but little attached either to human beings or the brute creation. His head was well formed, and his features very expressive for a negro.

The deposit was situated along both sides of the great falx of the dura mater and in that part of it nearest the falx; it extended for more than two inches along the line above mentioned, though it was not deposited in equal abundance nor continuously throughout that extent. Here and there it appeared as if but one or two fibres of the membrane were ossified, and in two places on one side of the falx, and one on the other, it was deposited in laminae of the size of the thumb nail and of two or three lines in thickness; its surface was rough and the edges sharp-pointed and serrated; the adhesions of the dura mater with the parts beneath was more extensive than is usual at these points; it produced a distinct indentation in the cerebral mass. Besides the above deposit, small depositions of calcarious matter were noticed in the substance of the lungs at various points.

Ossification of the fibrous, cartilaginous, and fibro-cartilaginous tissues, is very frequent in persons advanced in age; at this period the fibrous tissue of the sutures is changed and the sutures themselves become obliterated; the cartilages of the larynx, ribs, &c., are converted into bone; the symphysis pubis becomes agglutinated and similar changes occur in various others of these tissues, some of great detriment to the individual, and attended with considerable interruption of some of the vital functions. But when this deposition occurs at an early period of life, as in this case, and in parts where it does not most generally occur in the progress of life, it ceases to be a physiological phenomenon, and must be considered as the result of morbid action. That this alteration in early life is dependent upon some irritation is evident from various facts which have been observed, as for example the conversion of fibrous tissue in the neighborhood of fractured bone into bone, especially about the joints; and it has been perfectly ascertained that some exostoses have in reality no connection with the bone itself from whose surfaces they shoot up, but are formed exclusively from the periosteum which after an attack of chronic inflammation, attended with pain and swelling, has been converted into bone.

The irritation sufficient to give rise to a deposit of the character of the one now under consideration, did not occasion any serious symptoms, as is evident from the account of his general health; still there might have been a chronic and not very violent irritation at work; the irritability of temper he evinced would seem in some measure to corroborate the opinion. That there was some irritation preceding or subsequent to it, I think we are justified in believing from the more than natural adhesion which existed between the dura mater and tunica arachnoidea, though that irritation may not have been at any time sufficiently acute to have occasioned him any very great degree of uneasiness, or to have elicited the observation of any one.

The pressure which it must have exerted upon the brain, was at least equal to that which would have been occasioned by the depression to the same extent of a part of the inner table of the skull co-equal in size with it; now it is a well known and long established fact in surgery, that symptoms urgently demanding the use of the trephine are occasioned not unfrequently by a very slight fracture and depression of the vitreous table. The absence during the whole life of this individual of all the symptoms of compression seems to me, in view of the above fact, to be somewhat astonishing, and can only be accounted for by considering that it did not take place suddenly and all at the same time as in fracture; and by its gradual formation the brain was enabled to become accustomed to its presence, and for the same reason it did not occasion any considerable or acute symptoms of irritation.

That this kind of deposit in this situation, does sometimes occasion death, I have learned from examining some writers on pathology. Abercrombie, in his treatise on the brain, speaks of the occurrence of osseous developments in the dura mater as very common, most common in the falx major. Many cases of it are on record he says (but he does not mention whether they occurred at an advanced period of life or not), which did not appear to have produced any symptoms in the brain. In other cases however, he remarks, they seem to produce urgent symptoms, especially when they are in the form of sharp spicula or have acute angles which are so situated as to irritate the brain or its membranes. In a case of this kind by Laviard there was, in the broadest part of the falx, a small triangular piece of bone with sharp angles, and where the dura mater came in contact with these it was livid and discharged pus. In another by La Motte, which was connected with epilepsy, the bony spicula were directed against the pia mater; and in another by Van Swieten there was a large piece of bone in the substance of the brain. In a note, the American editor of the Dublin Dissector mentions that he has a specimen of ossification of the falx cerebri taken from a subject who died of apoplexy.

Most authors whom I have been able to consult, do not consider it as of very rare occurrence, nor that it is attended with inconvenience. Andral remarks that large cartilaginous or osseous plates are not unfrequently found in the meninges; the tentorium, he says, has been occasionally seen completely ossified. Dr. Baillie observes that the formation of plates of bone is one of the most common appearances of the dura mater. Dr. Gross has frequently observed them, and is inclined to consider that they originate rather in the subserous cellular tissue than in the membrane itself; he has most commonly noticed them in the falciform process, in masses from the size of the finger nail to that of a Spanish dollar, and says cases are given in which the whole of the dura mater of one hemisphere was ossified. Bichat says they are not rare and have their seat upon the internal surface of the membrane most frequently, but may happen in any part.

